# Multi-Lane Differential Variable Speed Limit Control via Deep Neural Networks Optimized by an Adaptive Evolutionary Strategy

**DOI:** 10.3390/s23104659

**Published:** 2023-05-11

**Authors:** Jianshuai Feng, Tianyu Shi, Yuankai Wu, Xiang Xie, Hongwen He, Huachun Tan

**Affiliations:** 1 School of Mechanical Engineering, Beijing Institute of Technology, Beijing 100081, China; 3120185238@bit.edu.cn (J.F.); hwhebit@bit.edu.cn (H.H.); 2 Intelligent Transportation Systems Centre, University of Toronto, Toronto, ON M5S 1A4, Canada; ty.shi@mail.utoronto.ca; 3 National Key Laboratory of Fundamental Science on Synthetic Vision, Sichuan University, Chengdu 610065, China; wuyk0@scu.edu.cn; 4 School of Information and Electronics, Beijing Institute of Technology, Beijing 100081, China; xiexiang@bit.edu.cn; 5 Advanced Research Institute of Multidisciplinary Sciences, Beijing Institute of Technology, Beijing 100081, China

**Keywords:** connected and autonomous vehicles, deep neural networks, evolutionary strategies, variable speed limits

## Abstract

In advanced transportation-management systems, variable speed limits are a crucial application. Deep reinforcement learning methods have been shown to have superior performance in many applications, as they are an effective approach to learning environment dynamics for decision-making and control. However, they face two significant difficulties in traffic-control applications: reward engineering with delayed reward and brittle convergence properties with gradient descent. To address these challenges, evolutionary strategies are well suited as a class of black-box optimization techniques inspired by natural evolution. Additionally, the traditional deep reinforcement learning framework struggles to handle the delayed reward setting. This paper proposes a novel approach using covariance matrix adaptation evolution strategy (CMA-ES), a gradient-free global optimization method, to handle the task of multi-lane differential variable speed limit control. The proposed method uses a deep-learning-based method to dynamically learn optimal and distinct speed limits among lanes. The parameters of the neural network are sampled using a multivariate normal distribution, and the dependencies between the variables are represented by a covariance matrix that is optimized dynamically by CMA-ES based on the freeway’s throughput. The proposed approach is tested on a freeway with simulated recurrent bottlenecks, and the experimental results show that it outperforms deep reinforcement learning-based approaches, traditional evolutionary search methods, and the no-control scenario. Our proposed method demonstrates a 23% improvement in average travel time and an average of a 4% improvement in CO, HC, and NOx emission.Furthermore, the proposed method produces explainable speed limits and has desirable generalization power.

## 1. Introduction

The steadily increasing number of population and economic activities has led to an increase in freeway congestion. Along this line, several dynamic traffic-management measures such as ramp metering [1,2], dynamic routing [3], and real-time travel information systems have been widely used for reducing congestion. One promising strategy is the use of variable speed limit (VSL) control [4,5,6], which not only harmonizes the speed within each lane and across different lanes but also increases the capacity of the freeway. Given the dynamic nature of freeway traffic systems, VSL is typically modeled as a feedback control problem. The controller automatically adjusts the speed limits according to real-time measurements of the freeway traffic state, and the objective is to achieve a desirable controlled variable, i.e., low bottleneck density and high-throughput rate. Göksu et al. [7] proposed a VSL controller, which utilized saturation feedback to ensure the integral input of traffic state to system stability. Some methods also considered integrating the ramp metering and variable speed limit control together to improve freeway traffic control performance [8,9].

One of the main challenges of implementing this type of control is that it needs to accurately predict the effect of the different speed limits of different lanes on traffic flow. This can be difficult to do, as traffic patterns are often complex and can be affected by a wide variety of factors. Additionally, different speed limits may not be effective in all situations and may even cause more congestion in some cases. It is also important to ensure that the different speed limits are able to contribute to congestion mitigation so that they can adjust their driving behavior accordingly.

In the machine learning context, reinforcement learning (RL) has been widely used for achieving feedback control autonomy [10,11]. RL has also effectively boosted the development of intelligent transportation system [12,13]. RL is a goal-oriented learning tool wherein the agent learns a policy to optimize a long-term reward by interacting with the environment. At each step, an RL agent receives the state feedback of the environment, and acts accordingly. The evaluative feedback about its performance in each step allows it to improve the performance of subsequent actions. In a control engineering context, RL can be viewed as a feedback control algorithm. Given the coherence between RL and VSL, researchers start to investigate reinforcement learning (RL) techniques for VSL control [14,15,16]. Several studies have reported the superior performance of RL techniques over traditional control approaches.

The success of modern RL systems is largely due to their combination with deep neural networks. With deep neural networks, RL agents are able to learn a meaningful state representation [17]. In general, deep reinforcement learning (DRL) is defined as the utilization of deep learning algorithms within RL. As DRL algorithms have performed amazing feats in recent years, there have been some attempts to apply DRL to VSL control [15,18]. Ke et al. [18] investigated using the transfer learning method to improve the transferability of deep reinforcement learning variable speed limits (VSL) control. For VSL control, DRL exhibit major difference from traditional RL methods. Their controllers are built upon deep neural networks which have the capacity to learn more complex representations of the environments. This expressive power allows for learning more optimal speed limits [15].

Despite the promising results of DRL, training the deep neural networks of DRL is still not an easy task. The algorithms for training DRL agents are highly sensitive and require a long learning process [19,20]. This issue mainly stems from the difficulties in defining a representative reward function for VSL agents. There are some recent works that use adversarial perturbed state observations to obtain the worst case reward in order to improve the robustness of the agent [21,22]. However, we need to know how the perturbations are generated. Essentially, the goal of traffic management is to reduce travel time and increase traffic flow capacity. However, in practice, the average travel time and total flow cannot be computed until all the vehicles have completed their routes, which causes the issue of delayed rewards. Handling delayed rewards is one of the most critical challenges in RL research [23,24]. The training through one-step agent–environment interaction is ineffective because the definition of which action induces rewards is ambiguous. This problem is often solved by reward engineering so that the reward is more related to the current action of the agent. However, reward engineering is a tedious process, which requires not only intensive parameter tuning but also specific domains. More importantly, the choosing reward may not necessarily lead to improvements of the freeway capacity.

Recent research has shown that evolutionary strategy (ES) algorithms can be used for tasks that are dominated by DRL algorithms [25]. In DRL algorithms, neural networks explore different actions in each step and these actions return rewards that are used to update the parameters of the network via backpropagation. Instead of using backpropagation, ES uses a “random search” approach to search the optimal neural networks parameters. It involves a number of agents acting in parallel using parameters sampled from a given distribution. Each agent acts in its own environment, and once it finishes a set number of episodes, the cumulative reward is returned to the algorithm as a fitness score. With this score, the parameter distribution moves toward that of the more successful agents. ES algorithms offer an attractive advantage when compared to DRL algorithms on VSL control tasks. The fitness score guiding the learning process is the overall return of the whole control period. As a result, the problem of the delayed reward for training deep neural networks can be readily solved with ES. In other words, we can directly use the total traffic flow or average travel time to train an ES agent [26,27].

In essence, evolution strategy (ES) is a nature-inspired direct search and optimization method which uses mutation, recombination, and selection applied to a population of individuals in order to evolve iteratively better and better solutions. Several types of ES algorithms have been proposed in the literature. The difference of these models mainly lies in how they represent the population and how they perform mutation and recombination. For example, Salimans et al. [25] already showed that deep neural networks learned by a special natural evolution strategy (NES) can achieve competitive results in video games and robotic control. NES updates the search distribution in the direction of higher expected fitness using the gradient induced from fitness score. Afterwards, Such et al. [28] also showed that the gradient-free approach genetic algorithms (GAs) are also a competitive alternative for training deep neural networks on DRL tasks.

The version of ES we use in this work belongs to the adaptive evolution strategies. We use the covariance matrix adaptation evolution strategy (CMA-ES) [29], which represents the population by a full-covariance multivariate Gaussian. The recombination process of CMA-ES refers to selecting a new mean value for the multivariate Gaussian. Mutation amounts to adding a random vector with a zero mean. The dependencies between different individuals within the population are represented by a covariance matrix. The covariance matrix adaptation (CMA) is to update the covariance matrix of this distribution. We choose CMA-ES since it is known to converge faster than other ES, and can automatically adapt the learning step and noise distribution. We evaluate the proposed CMA-ES optimized deep neural network controllers on a simulated freeway traffic with recurrent bottlenecks, in which distinctive and dynamic speed limits among lanes are allowed. The main contribution of our paper can be summarized as the following:

- We propose a multi-lane differential variable speed limit control framework which can better handle the delayed reward.
- We propose the CMA-ES based VSL algorithm to improve the robustness of our control framework, which needs less parameter tuning. The CMA-ES also learns explainable control policy.
- Various experimental results show that CMA-ES agent is better than non-evolutionary search (NES) and deep reinforcement learning (DRL) agents in the real-world freeway network with on-ramp and off-ramp roads.

## 2. Problem Statement

**Figure 1:**
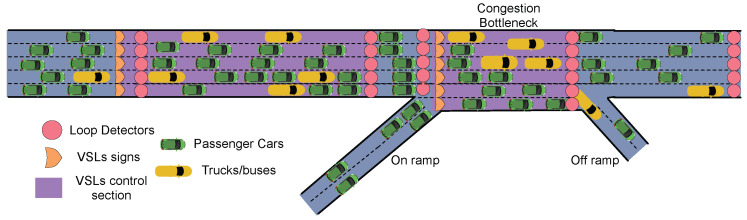
The freeway section has an on-ramp and an off-ramp. There is a recurrent bottleneck caused by conflicts between the inflow in the on-ramp and the outflow in the main lane. There are several traffic detectors. The VSL control in both the bottleneck and its upstream section is permitted. Different lane speeds in each lane are allowed.

The freeway section considered in this paper is given in Figure 1. The freeway section in Figure 1 is composed of multiple on-ramp and an off-ramp lanes.As we can see, the interference between vehicles mainly happens in the merging area between the inflow of on-ramp roads and the outflow of the mainstream. The conflicts cause further speed reductions in the merging area, contributing to the creation of a generalized bottleneck.

Following the framework of our previous work [15], different speed limits across different lanes in the same control freeway section are allowed in this work. The benefits of differential variable speed limits (DVSL) among lanes are limited by driver compliance. Therefore we assume that the DVSL system is running on a connected and automated vehicle (CAV) environment, in which the infrastructure can send commands to the vehicles, and vehicle speed commands or speed limits are automatically enforced.

The DVSL control system in Figure 1 includes three sections: (1) the upstream of the bottleneck in which the outflow is controlled by adjusting the posted speed limits (purple); (2) the bottleneck with congestion in which the speed limits can change (purple); (3) a speed modulation section that allowed vehicles to accelerate from low speed adjust its speed between two controlled sections (dark blue). Traditional systems only change the speed of the upstream. We also implement VSL signs in the recurrent bottleneck. The function of upstream speed limit control is to adjust the outflow to the bottleneck and prevent the capacity drop of the downstream bottleneck. The speed limits in the bottleneck control the congestion formation and dissipation. If the congestion is presented in the bottleneck, a lower speed limit would be more desirable as it harmonizes the driving speed of all vehicles. On the other hand, a higher speed limit can also allow the congestion to dissipate faster.

The DVSL control problem is modeled as a temporal decision process. In each time step, the DVSL agent determines the speed limits of each controlled lane according to the occupancy rate reported by the loop detectors. The mapping between the traffic state and speed limits is represented by a policy $\pi_{\theta }$ parameterized by θ. The speed limits $v_{t}$ at time point *t* can be calculated by

(1) $$v_{t}=\pi_{\theta }\left(s_{t}\right),$$

where $v_{t}\in R^{n}$, *n* is the number of the controlled lanes, and the traffic state observation $s_{t}\in R^{m}$ at time point *t*, m, is the number of the loop detectors in this system.

The goals of VSL control can be various. A main targeted impact of VSL is to enhance traffic safety; other targets include improvement of traffic flow efficiency, such as in the sense of reduced travel times and environmental (noise, pollution) benefits. The main target of this study is the efficiency of the freeway section. The efficiency can be measured by the system travel time. In real-world applications, it is impractical to calculate all the vehicles’ travel time. However, we can use the loop detectors in the entry and exit of the freeway to calculate the discrete representation of system travel time. The discrete-time representation of system travel time (TTT) over the control time horizon T can be calculated by

(2) $$TTT=\eta \sum_{t=1}^{T}N\left(t\right)=N\left(0\right)+\eta \sum_{t=1}^{T}\left(N^{in}\left(t\right)-N^{out}\left(t\right)\right),$$

where N(t) is the total number of vehicles in the network at time *t*. η is the time duration. $N^{in}\left(t\right)$ and $N^{out}\left(t\right)$ are the received and exit flow, respectively.

N(0) in Equation (2) is independent of the control measures taken in the freeway, hence the minimization of system travel time is equivalent to searching for policy $\pi_{\theta }$ that maximizes the following quantity

(3) $$\max_{\pi_{\theta }}-\sum_{t=1}^{T}\left(N^{in}\left(t\right)-N^{out}\left(t\right)\right),$$

In this study, the number of vehicles $N^{in}\left(t\right)$ entering the freeway system at each time step from both upstream mainline and on-ramp are recorded. The number of vehicles $N^{out}\left(t\right)$ leaving the freeway system at downstream mainline and off-ramp is also recorded. We model policy $\pi_{\theta }$ by a deep neural network, and its parameter θ is optimized by CMA-ES [29], in which the feature is encoded using three-layer neural networks. The neural network structure and optimization method will be discussed in the next section.

Given the large amount of mathematical notation employed in the remainder of this article, we have included a brief summary of the nomenclature in Nomenclatures.

## 3. Proposed Method

In this section, we will introduce how to integrate the CMA-ES introduced above to optimize deep neural networks to generate variable speed limits. The policy $\pi_{\theta }$ is built upon deep neural networks parameterized by θ. Due to the driver compliance issue, a continuous speed limit is not feasible to post in a variable speed sign. However, it is more convenient to design a deep neural network with continuous outputs. To avoid this contradiction, we first generate a continuous neural networks output of $a_{t}\in R^{n}$ at time steps *t* given observation $s_{t}$ by

(4) $$a_{t}=f_{\theta }\left(s_{t}\right),$$

**Figure 2:**
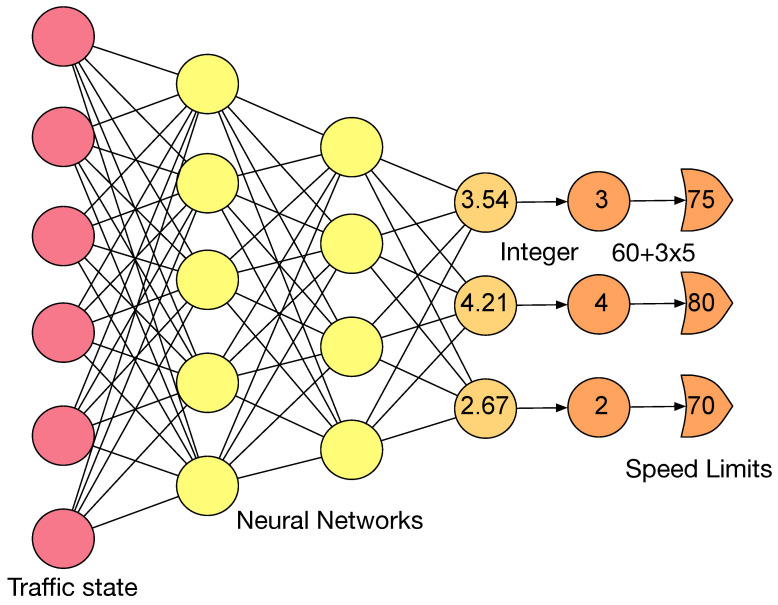
The illustration of the policy $\pi_{\theta }$. It uses deep neural networks to generate variable speed limits from traffic state.

$f_{\theta }$ is a neural network parameterized by θ. $s_{t}\in R^{m}$ is the real-time traffic states reported by *m* loop detectors. The value of $a_{t}$ is clipped between [0,1,⋯,M). *M* is the number of feasible speed limits. Then the discrete action $\hat{a_{t}}$ is obtained by choosing the integer parts of $a_{t}$. Obviously, $0\leq \hat{a_{t}}\leq M-1$. Then the speed limits $v_{t}$ is calculated by $v_{t}=v_{0}+I\hat{a_{t}}$. $v_{0}\in R^{n}$ is the minimum value of the speed limit, *I* is the integer multiples, the maximum value of speed limits is $v_{0}+I\left(M-1\right)$. For example, if the feasible speed limits is [60 km/h, 65 km/h, ⋯, 120 km/h], then $v_{0}$ = 60 km/h, *I* = 5 km/h, and M=13. Figure 2 gives an illustration of the policy $\pi_{\theta }$.

The next step is to obtain the optimal parameters θ of deep neural networks that maximize the reward $J_{\theta }=-\sum_{t=1}^{T}\left(N^{in}\left(t\right)-N^{out}\left(t\right)\right)$. There have been various kinds of DRL algorithms for learning parameters θ, the common theme is to use backpropagation, which involves a gradient approximation of parameter θ. However, optimizing θ alone for high reward by the gradient can cause the model to become stuck in local optima, and as a result, the agent may fail to learn appropriately. This motivates us to explore the gradient-free evolutionary algorithms to optimize θ. More specifically, in this work we use the covariance matrix adaptation evolution strategy (CMA-ES).

ES is broadly based on the principle of biological evolution, namely the repeated interplay of variation (via recombination and mutation) and selection. In each generation, some individuals are selected to become the parents in the next generation based on their fitness score. For VSL control, the fitness score is the system travel time term $J_{\theta }=-\sum_{t=1}^{T}\left(N^{in}\left(t\right)-N^{out}\left(t\right)\right)$. θ represents the bias and weights of the neural networks. The next generation are generated by the variation of the parents. The individuals of the new generation are working on the VSL control environment in parallel. The variation rule of the CMA-ES is given below:

(5) $$\theta_{k}^{g+1}={\left(\theta \right)}_{\mu }^{g}+\alpha^{g}\times N\left(0,C^{g}\right),$$

where $\theta_{k}^{g+1}$ is the *k*-th parameter of g+1-th generation, $\alpha^{g}>0$ is the step size, $C^{g}$ is the covariance matrix of the noise, ${\left(\theta \right)}_{\mu }^{g}=\frac{1}{\mu }\sum_{i\in I_{sel}^{g}}\theta_{i}^{g}$ represents the center of mass of the selected individuals of generation. The selection set $I_{sel}^{g}$ is determined by the fitness score $J_{\theta }$. $|I_{sel}^{g}|=p$, For each evolutionary generation, we generate *K* individuals, and evaluate them on the same DVSL task to obtain their fitness score. The *p* fit individuals with larger $J_{\theta }$ are selected as members of $I_{sel}^{g}$.

CMA-ES provides an adaptive mechanism to update covariance matrix $C^{g}$ and updates step size $\alpha^{g}$. First, the evolution path $p_{c}^{g+1}$ is calculated by

(6) $$p_{c}^{g+1}=\left(1-c_{c}\right)p_{c}^{g}+\sqrt{pc_{c}\left(2-c_{c}\right)}B^{g}D^{g}{\left(\theta \right)}_{\mu }^{g+1},$$

$c_{c}$ is the update step for evolution path, the column of $B^{g}$ represent normalized eigenvectors of $C^{g}$, $D^{g}$ is a diagonal matrix whose elements are the square roots of the eigenvalues of $C^{g}$. Then $C^{g+1}$ is updated by

(7) $$C^{g+1}=c_{cov}C^{g}+\left(1-c_{cov}\right)p_{c}^{g+1}{\left(p_{c}^{g+1}\right)}^{T},$$

$c_{cov}$ is the update step for covariance matrix.

Next step is to adapt the global step size $\alpha^{g}$, the evolution path $p_{\alpha }^{g+1}$ is calculated by

(8) $$p_{\alpha }^{g+1}=\left(1-c_{\alpha }\right)p_{\alpha }^{g}+\sqrt{pc_{\alpha }\left(2-c_{\alpha }\right)}B^{g}D^{g}{\left(\theta \right)}_{\mu }^{g+1},$$

$c_{\alpha }$ is the update step for $p_{\alpha }$. The length of the evolution path determines the step size for generation g+1.

(9) $$\alpha^{g+1}=\alpha^{g}\cdot \exp\left(\frac{1}{d_{\alpha }}\frac{∥p_{\alpha }^{g+1}∥-x_{n}}{x_{n}}\right),$$

where $x_{n}$ is the update parameter for $p_{\alpha }$, $d_{\alpha }>1$ is a dumping parameter.

In summary, CMA-ES estimates the variation of neural network individuals through incremental adaption along evolution paths and contains information about the correlation between consecutive updates. With CMA-ES, we can use an adaptive gradient-free approach to update the neural network controller for VSL control task. In this work, we use the open-source package pycma (https://github.com/CMA-ES/pycma, accessed on 5 September 2021) with distributed workers as the optimization tool for our deep neural networks. Each worker operates independently, and samples a perturbation $\epsilon_{i}$ from a normal distribution with a mean of 0 and a standard deviation σ. Then, it computes the returns based on the throughput $F_{i}$, which is the negative sum of the difference between the inflow and outflow of vehicles for each time step *t*. The complete Algorithm 1 is summarized below:
**Algorithm 1** Adaptive Evolutionary Strategy for VSL control 1:Initialize reward function and neural network’s parameters, noise standard deviation σ, initial policy parameters θ 2:**for** Every time step t = 0, 1, 2… **do** 3:    **for** Each worker i = 1, …, n **do** 4:        Sample the perturbation $\epsilon_{i}∼N\left(0,I\right)$ 5:        Compute returns based on the throughput $F_{i}=-\sum_{t=1}^{T}\left(N^{in}\left(t\right)-N^{out}\left(t\right)\right)$ 6:    **end for** 7:    Send all scalar returns $F_{i}$ to every worker 8:    **for** each worker i=1,…,n **do** 9:        Reconstruct all perturbations $\epsilon_{j}$ for j=1,…,n using random seeds10:        update $\theta_{t+1}$ based on $\theta_{k}^{g+1}={\left(\theta \right)}_{\mu }^{g}+\alpha^{g}\times N\left(0,C^{g}\right)$11:    **end for**12:**end for**

## 4. Experiments

In this section, we mainly conducted experiments on a simulated freeway section built by SUMO to evaluate the effectiveness of CMA-ES-based DVSL control.We set the initial control time horizon T as 60, the incident time as 0, and the incident length as 0. We selected the batch size as 32. The noise standard deviation is set as 1, we use the *normalized columns initializer* for initialization [30]. The learning rate is set as 0.1. We run the experiment 100,000 times.The terminal is activated when it exceeds the total simulation step (18,000); meanwhile, if all vehicles have left the network or a collision happens, the terminal will also be intrigued.

### 4.1. The Simulated Freeway Section

**Figure 3:**
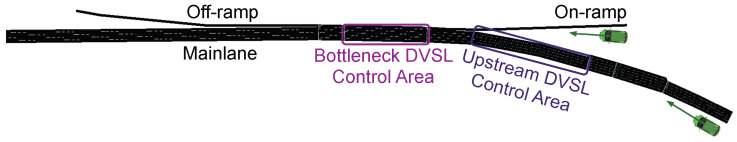
The topology of the simulated freeway section.

**Figure 4:**
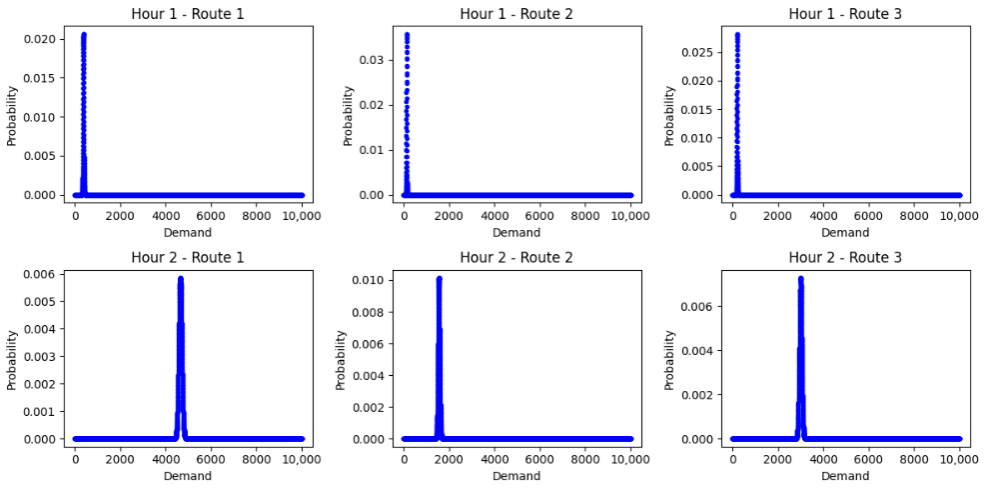
Demand distribution for different routes in different time periods.

We use the open-source software SUMO to implement our experiments. The software supports setting the speed limits for each lane using its API, the Traffic Control Interface (TraCI) package. An 874.51 m freeway section with on- and off-ramps of I405 north bound in California, USA is selected. The original speed limits for the main-lane of this section are 65 mile/h and for the on- and off-ramps are 50 mile/h. Figure 3 illustrates the topology of the simulated freeway in SUMO. The travel demand of this freeway can be categorized into three routes: (1) from main-lane to main-lane (M2M), (2) from main-lane to off-ramp (M2Off), and (3) from on-ramp to mainline (On2M). We simulate 2 h of traffic for this freeway section. In the first hour, the hourly demand of these three routes is modeled as Poisson distribution with average values of 375 (M2M), 125(M2Off), and 200(On2M), respectively. In the second hour, the traffic turns to congestion, and the average values become 4650, 1550, and 3000, respectively. The depart lane of the vehicles are randomly set according to a uniform distribution. Passenger cars with a length of 3.5 m and truck/bus with a length of 8 m are selected as vehicle types in the simulated traffic stream. The combination of vehicles is generated randomly according to probability [0.85, 0.15].The demand distribution probability can be visualized as in Figure 4.

Different vehicles exhibit various and distinct driving behaviors in the real world. To account for this heterogeneity, we randomly assigned half of the vehicles with the “Krauss” car-following model, and the other half with the “IDM” car-following model [31]. The SUMO default “LC2013” model was used as the lane-change model for all vehicles.

We explore two critical characteristics of drivers. The first pertains to their desired speed with respect to speed limits and can be expressed through the “speedFactor” variable, which can also be modeled using a normal distribution. For DVSL agent training, we set the speedFactor to “normc(1,0.1,0.2,2)”, indicating that the speedFactor has a mean of 1 and a standard deviation of 0.1, and ranges from 0.2 to 2.

The second property we investigate is the propensity of vehicles to execute rule-based lane changes to increase their speed. To capture this, we introduce a “speedgen” variable that corresponds to the “lcSpeedGain” attribute. When training VSL agents, we set the “lcSpeedGain” attribute to 1, indicating that the vehicle is more likely to change lanes to achieve higher speeds. A higher “lcSpeedGain” value suggests that the vehicle is more inclined to change lanes to gain speed.

To collect state variables for DVSL control, we place loop detectors in the freeway section. We set up 22 loop detectors around two control areas to monitor the traffic state. The occupancy rate of these 22 detectors, which reflects the congestion level of the corresponding areas, serves as input $s_{t}$ for the deep neural networks. The variable speed limits range from 40 to 75 miles per hour and change every five minutes. We also use SUMO simulation software in conjunction with the Handbook Emission Factors for Road Transport (HBEFA)4.1 [32] to model emissions, obtaining the CO, HC, NOx, and PMx.

### 4.2. Comparison Results

The total travel time index given in Equation (2) is used as the fitness score for ES-based algorithms and the reward function for the DVSL-based control agent. The DVSL controller of all the methods is built upon a three-layer deep neural network. The first layer has 60 hidden neurons, and the second layer has 30 hidden neurons. The ReLU function is used as the activation function for the hidden layer, the Sigmoid function is used the activation function for the output layer. For the evolutionary strategy-based model, each generation has 8 individuals, and 1000 episodes are used to optimize the neural networks. For DDPG, we use 1000 episodes to train the DRL-based controller.

We compare CMA-ES based DVSL control with the following baseline methods, which include:

- No control: The baseline without any control.
- DDPG-based DVSL control (DDPG): A DRL-based DVSL control method proposed in [15].
- Deep Q networks (DQN): we add another baseline DQN which is developed based on [17] and we use the same state and reward as DDPG to make a fair comparison. We add this baseline to compare discrete control action and continuous control action in variable speed limit control problems.
- Natural ES-based DVSL control (ES): the DVSL controller optimized by the natural ES-based algorithm, proposed in [25].

**Figure 5:**
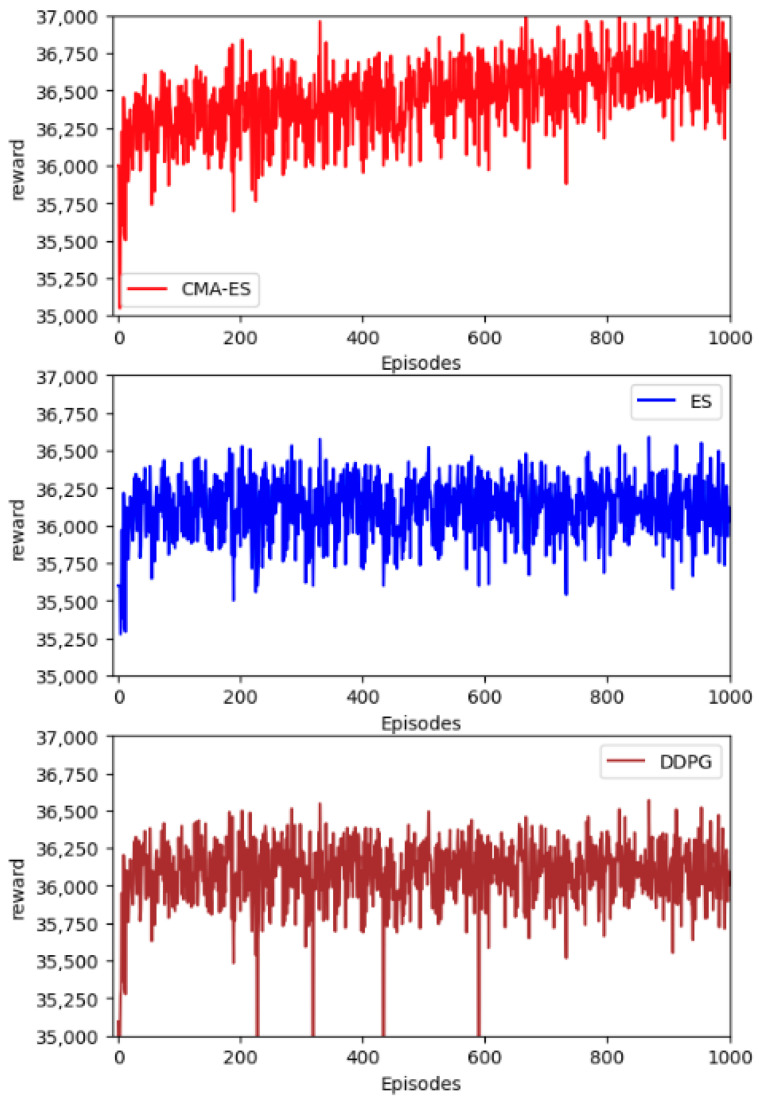
The reward performance comparison for different learning methods.

To compare the reward behavior over the learning process for all used learning-based algorithms, we visualized the plot for ES, CMA-ES, and DDPG in the following Figure 5. We can find that our proposed CMA-ES algorithm can achieve the highest reward compared to the ES and DDPG methods. Meanwhile, it is more stable compared to the DDPG method.

Table 1 compares the best performance of different methods. The results show that CMA-ES outperforms both ES- and DDPG-based methods in terms of average travel time, emission, and safety. CMA-ES can reduce average travel time by 18.12%, CO emission by 12.76%, and HC emission of the freeway by 11.11%. Most importantly, it can reduce emergency brake use by 2%, which indicates an improvement for safety. Both ES and CMA-ES outperform the DDPG-based controller, indicating that evolutionary strategies are more suitable for VSL control tasks compared with the DRL method. All the methods cannot reduce the NOx and PMx emissions. Interestingly, the DDPG-based controller even increases NOx and PMx emissions, though it also improves the efficiencies of the freeway. We can also find that continuous control action space design (i.e., DDPG) will be better than discrete control action space design (i.e., DQN).

**Figure 6:**
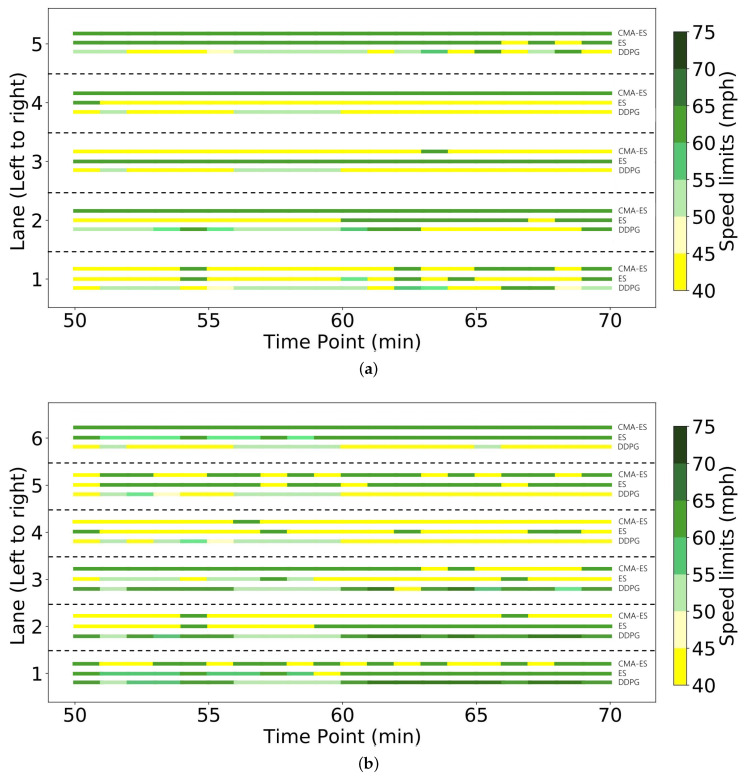
Visualization of the speed limits given by CMA-ES, ES, and DDPG-based controllers. (**a**) The speed limits given by different methods for upstream section from the 50th minute to the 70th minute. (**b**) The speed limits given by different methods for bottleneck section from the 50th minute to the 70th minute.

Comparative studies between ES and DRL on video games have shown that they exhibit different behavior patterns. ES’s behavior is dominated by the prior action distribution, whereas DRL tends to react to the real-time state changes [33]. To study the behaviors patterns of DRL and ES on the VSL task, Figure 6 plots the speed limits given by CMA-ES, ES, and DDPG based algorithms. Obviously, the behaviors of DDPG in Figure 6 look more random, whereas CMA-ES produces more regular patterns. The behavior of ES seems like an intermediate point between CMA-ES and DDPG. The reason might be that ES uses both gradient information and evolutionary strategies. The behaviors of CMA-ES can be viewed as the combination of static rules and dynamic feedback control. The most left lane of the upstream and bottleneck sections are ruled by the highest speed limits of 75 mph. The CMA-ES are learned to set those lanes as overtaking lanes. This accords with the simple traffic rule as the experiments are running on a right-driving environment. In other lanes, the CMA-ES dynamically switches the speed between the highest 75 mph and the lowest 40 mph according to real-time traffic state. The CMA-ES seldom uses the speed limits between 40 and 75. Compared with the upstream control section, the speed limits of the bottleneck are more often set to the lowest value. Obviously, the speed limits produced by CMA-ES are explainable and easy to understand. Moreover, this type of behavior leads to superior performance compared with the random behavior of DDPG. Additionally, the speed limits produced by the CMA-ES algorithm can be viewed as a combination of static rules and dynamic feedback control, making them more interpretable. For instance, the CMA-ES algorithm learns to set the most left lane of the upstream and bottleneck sections as overtaking lanes with the highest speed limit of 75 mph, which aligns with the simple traffic rules in a right-driving environment. On the other hand, DDPG produces speed limits that do not align with any discernible traffic rules, making them more difficult to interpret.

### 4.3. Assessing Generalization

For this research, the ES and DRL agents were exclusively trained in a simulation environment. However, a crucial question arises: how can a DVSL agent leverage simulation to perform practical tasks in the real world? Additionally, how can we extend its performance to novel scenarios that differ from the simulation? Traffic simulation presents a challenge, since even the most sophisticated simulators cannot fully replicate real traffic flow dynamics and uncertainty. We can evaluate the agents’ generalization abilities by subjecting them to test environments with varying attributes from the ones they were trained on. This ability to generalize provides us with evidence of the agents’ performance in a different environment, such as a real-world implementation with different attributes from the simulated environment. The simulation environment employed in this study is expected to be equipped with advanced CAV techniques, with CAVs adhering to the recommended speeds. Therefore, we are intrigued by the performance variations of DVSL control concerning critical attribute changes introduced by CAV techniques. The first important attribute is the driver’s minimum time headway. During training, this value τ is set to 1 s. We evaluate the agents on environments with τ=0.25 and τ=0.5.

Table 2 gives the performance of different methods with different τ. It shows that highly developed CAV with a very low reaction time is beneficial for freeway traffic. It is obvious that smaller τ leads to lower travel time and less emission. DDPG fails to improve the freeway performance when τ is lower to 0.25. Both ES and CMA-ES can still reduce the travel time and emission under an environment with lower τ. The second change brought by CAV technique might be that the vehicles are more likely to drive under the speed limits. This can be simulated lower down the standard deviation $\sigma_{s}$ of “speedFactor”. We evaluate the DVSL models with $\sigma_{s}=0.05$ and $\sigma_{s}=0.075$.

Table 3 gives the results of different methods with different deviation $\sigma_{s}$. The lower deviation will lead to lower throughput and higher emission for the freeway. The higher deviation value makes the driving speed more diverse, which would allow more gaps for merging vehicles, and improves the efficiency of merging area. In this case, the DDPG-based DVSL agent fails to improve the performance of the freeway. ES outperforms CMA-ES when $\tau_{s}$ is lower, though CMA-ES outperforms ES when $\tau_{s}=0.1$. The results show that the efficiency of traffic will be improved by a more diverse driving style. Therefore, it might be desirable to add some random driving mechanism to the CAVs as this will lead to systematic benefits.

**Figure 7:**
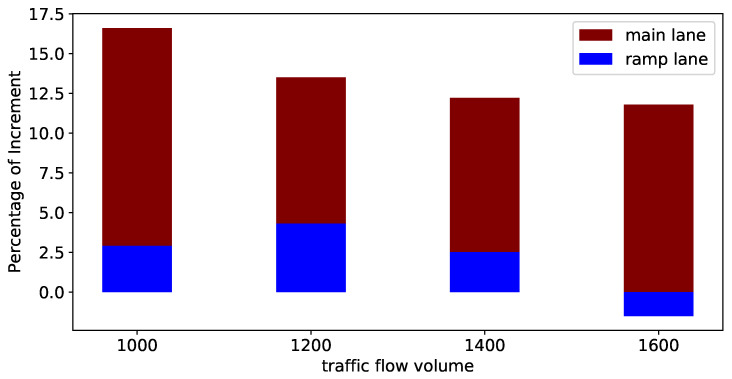
The performance on mainline and ramp lane.

In order to further demonstrate the robustness of our model to different flow rates, we analyzed our model’s performance in comparison to non-control performance. We measured the percentage of improvement in average travel time corresponding to different flow rates. As shown in Figure 7, we can see that in comparison to the non-control framework, our model demonstrates robustness when the flow volume increases on the main lane and ramp lanes. The red histograms represent the main lane while the blue histograms represent the ramp lane. We can observe that when the traffic flow increasea, the percentage of increment of main will decrease; however, we can observe that given an extremely high volume, i.e., 1600, the ramp lane efficiency will decrease. Given a very high traffic volume, the model will learn to sacrifice some travel efficiency on ramp lanes in order to increase system-level efficiency.

## 5. Conclusions and Discussion

In this paper, we have proposed a gradient-free and adaptive evolutionary strategy for multi-lane differential variable speed limit control. In order to learn the dependence between traffic state and optimal action, the deep neural networks were used to learn the representation of the traffic state. Our solution outperforms the DRL-based solutions in terms of improvements in freeway capacity and emission reduction. The learned speed limits are explainable and can be generalized to environment with different properties.

The proposed method has demonstrated promising results in the task of multi-lane differential variable speed limit control, outperforming deep reinforcement learning-based approaches, traditional evolutionary search methods, and the no-control scenario. However, one limitation of the proposed work is that it requires a significant amount of computational resources, as it relies on a large number of parallel workers to efficiently optimize the policy parameters. Future work could explore ways to reduce the computational cost of the proposed method while maintaining its effectiveness.

Several interesting questions stem from our paper both theoretically and practically, which we plan to study in the future. We aim to extend the approach to large freeway networks and a broader set of dynamic events, such as adverse weather and traffic incidents, in the future. Another interesting direction we plan to study is the incorporation of more advanced traffic-control strategies. In this paper, the most basic neural network architecture and adaptive evolutionary strategy are used. We believe that more systematic research of architectures such as graph convolutional networks [34] and optimization strategies may provide improvements in control performance. Furthermore, it is also interesting to study the cooperation effect of connected automated vehicles [35] and the infrastructure.

## Figures and Tables

**Table 1:** Average performance of different methods. The best controllers for each index are shown in boldface.

Method	$-\sum_{t=1}^{T}\left(N^{\mathrm{in}}\left(t\right)-N^{\mathrm{out}}\left(t\right)\right)$	Average Travel Time (s)	CO (kg)	HC (kg)	NOx (kg)	PMx (kg)	Num of Emergency Brake
No control	−183	81.36	47.80	0.27	0.94	0.047	610
DDPG [15]	−155	80.17	46.36	0.27	0.97	0.048	500
DQN [17]	−177	80.87	46.98	0.26	0.97	0.048	580
ES [25]	−138	69.74	42.41	0.25	0.94	0.047	450
CMA-ES	**−106**	**66.61**	**41.70**	**0.24**	0.94	0.047	**440**

**Table 2:** Average performance of different models with different τ. The best controllers for each index are shown in boldface.

Method	$-\sum_{t=1}^{T}\left(N^{\mathrm{in}}\left(t\right)-N^{\mathrm{out}}\left(t\right)\right)$	Average Travel Time (s)	CO (kg)	HC (kg)
τ=0.5				
No control	−148	72.10	45.08	0.26
DDPG	−146	72.21	43.23	0.25
DQN	−147	72.41	44.13	0.24
ES	−132	**61.41**	**40.14**	**0.24**
CMA-ES	**−130**	64.03	41.63	0.25
τ=0.25				
No control	−148	63.60	42.25	0.25
DDPG	−143	69.38	43.16	0.25
DQN	−145	67.83	43.12	0.25
ES	**−119**	62.61	41.50	0.25
CMA-ES	−129	**61.42**	**41.32**	**0.24**

**Table 3:** Average performance of different models with different $\sigma_{s}$. The best controllers for each index are shown in boldface.

Method	$-\sum_{t=1}^{T}\left(N^{\mathrm{in}}\left(t\right)-N^{\mathrm{out}}\left(t\right)\right)$	Average Travel Time (s)	CO (kg)	HC (kg)
$\sigma_{s}=0.05$				
No control	−192	83.65	50.48	0.29
DDPG	−201	83.51	48.96	0.29
DQN	−199	83.98	48.13	0.29
ES	**−124**	**67.60**	**43.30**	**0.25**
CMA-ES	−147	73.91	46.80	0.27
$\sigma_{s}=0.075$				
No control	−170	74.33	45.92	0.26
DDPG	−156	78.08	45.60	0.26
DQN	−167	98.91	46.62	0.27
ES	**−141**	**66.23**	**42.14**	**0.25**
CMA-ES	−152	69.90	44.26	0.26

## Data Availability

Not applicable.

## Nomenclatures

The following Nomenclatures are used in this manuscript:

Symbol	Description
$v_{t}$	The speed limits at time point *t*
$s_{t}$	traffic state observation at time point *t*
$a_{t}$	continues neural networks outputs at time point *t*
$f_{\theta }$	a neural network parameterized by θ
*M*	number of feasible speed limits
$C^{g}$	covariance matrix
$p_{c}^{g+1}$	evolution path
$B^{g}$	normalized eigenvectors
$c_{cov}$	update step for covariance matrix
N(t)	total number of vehicles in the network at time *t*
